# Report of Treasurer of World’s Columbian Dental Congress in Full from March 23d, 1891, to July 23d, 1895

**Published:** 1895-11

**Authors:** 


### REPORT.

### Report of Treasurer of World's Columbian Dental Congress in Full from March 23d, 1891 to July 23d, 1895.

RECEIPTS.

| | |
|---|---|
| American Dental Asso. | $1,000.00 |
| Illinois State Dental Soc. | 321.00 |
| Iowa State Dental Soc. | 100.00 |
| Southern Dental Asso. | 200.00 |
| Washington City Den.Soc. | 50.00 |
| Alabama | 130.00 |
| Arizona | 20.00 |
| Arkansaw | 20.00 |
| Austria | 10.00 |
| California | 548.00 |
| Canada | 20.00 |
| Connecticut | 220.00 |
| China | 30.00 |
| Colorado | 40.00 |
| Chili | 10.00 |
| Cuba | 10.00 |
| Delaware | 50.00 |
| District of Columbia | 190.00 |
| England | 50.00 |
| France | 100.00 |
| Florida | 20.00 |
| Georgia | 125.00 |
| Germany | 10.00 |
| Hawaii Islands | 40.00 |
| Illinois | 3,802.00 |
| Iowa | 460.00 |
| Indiana | 355.00 |
| Italy | 10.00 |
| Kentucky | 120.00 |
| Kansas | 130.00 |
| Louisiana | 60.00 |
| Maryland | 250.00 |
| Massachusetts | 395.35 |
| Mississippi | 40.00 |
| Michigan | 245.00 |
| Mexico | 20.00 |
| Maine | 190.00 |
| Minnesota | 405.00 |
| Montana | 10.00 |
| Missouri | 715.00 |
| Miscellaneous | 11.70 |
| Nebraska | 180.00 |
| New Jersey | 477.50 |
| New York | 1,515.30 |
| North Carolina | 210.25 |
| New South Wales | 30.00 |
| North Dakota | 40.00 |
| New Hampshire | 10.00 |
| Ohio | 620.00 |
| Oregon | 20.00 |
| Pennsylvania | 840.30 |
| Paraguay | 20.00 |
| Rhode Island | 25.00 |
| Russia | 30.00 |
| South Dakota | 10.00 |
| South Carolina | 130.00 |
| Scotland | 20.15 |
| Switzerland | 20.00 |
| South America | 10.00 |
| Spain | 20.00 |
| Tennessee | 290.00 |
| Texas | 170.00 |
| Vermont | 120.00 |
| Virginia | 100.00 |
| Washington | 80.00 |
| West Virginia | 10.00 |
| Wisconsin | 150.00 |
| AmericanCollegeDen.Sur. | 10.00 |
| Baltimore College | 10.00 |
| Chicago College | 10.00 |
| Columbia University | 10.00 |
| Louisville Dental College | 20.00 |
| New York Dental College | 10.00 |
| Ohio Dental College | 10.00 |
| University of Buffalo | 10.00 |
| University of California | 10.00 |
| University of Minnesota | 10.00 |
| Univ. Western Reserve | 10.00 |
| Wilmington Den. Mfg. Co. | 40.00 |
| Philadelphia College | 10.00 |
| | $15,851.55 |
| Amount received from Columbia Nat'l Bank | 286.06 |
| Donations of Executive Committee | 3,600.00 |
| | $19,737.61 |

DISBURSEMENTS.

| | |
|---|---|
| General Finance Committee, L. D. Shepard | $ 926.25 |
| Executive Committee Secretary Expenses | 3,018.84 |
| Treasurer's Expenses | 583.59 |
| Treasurer's Bond | 100.00 |
| Secretary General's Expenses | 1,299.48 |
| State Conference Committee, J. Taft | 218.87 |
| Registration Committee, Fred A. Levy (per G. C. Brown) | 34.00 |
| Invitation Committee, W. C. Barrett | 16.95 |
| Clinics Committee, C. F. W. Bodecker and S. H. Guilford | 80.35 |
| Biology Committee, R. R. Andrews | 18.15 |
| Nomenclature Committee, G. V. Black | 31.00 |
| History Committee, J. Taft | 1,523.57 |
| Membership Committee, E. Noyes | 13.50 |
| Publication of Transactions Committee, A. W. Harlan | 4,529.95 |
| Donations by Executive Committee, Traveling Expenses | 3,600.00 |
| Woman's Department | 307.00 |
| Dental Manufacturing Co., S. S. White | 121.80 |
| Medals | 720.00 |
| Buttons and Badges | 257.63 |
| Club House | 1,530.00 |
| Banquet—Deficit | 274.60 |
| Refunded Memberships | 50.00 |
| Exchange | 6.36 |
| | $19,261.89 |
| Paid by Columbia National Bank | 286.06 |
| Unpaid by Columbia National Bank | 127.09 |
| | $19,675.04 |
| Balance, July 23d, 1895, in Merchants and Loan Trust Co. Bank. | 62.57 |
| | $19,737.61 |

(Signed) JOHN S. MARSHALL,
Treasurer.

